# Stries angioides et pseudoxanthome élastique

**DOI:** 10.11604/pamj.2015.20.250.6526

**Published:** 2015-03-16

**Authors:** Siham Chariba, Rajae Daoudi

**Affiliations:** 1 Service d'Ophtalmologie, Hôpital des Spécialités, Rabat, Maroc

**Keywords:** Stries angioïdes, pseudoxanthome élastique, ophtalmologie, angioid streaks, elastic pseudoxanthoma, ophtalmology

## Image in medicine

Il s'agit d'un patient âgé de 30 ans ayant un pseudoxanthome élastique, chez qui l'examen ophtalmologique de routine a retrouvé une acuité visuelle à 10/10ème avec au fond d'œil des lignes radiaires sombres partant de la papille aux deux yeux. A l'angiographie à la fluorescéine ces lignes sont hyper fluorescentes de manière inhomogène. L'association avec le pseudoxanthome élastique nous confirme qu'il s'agit de stries angioïdes. Cet aspect de stries angioïdes peu être pris a tord pour des vaisseaux choroïdiens ou rétiniens mais l'examen du fond d'œil et l'angiographie permettent de les distinguer. Cet aspect peut également prêter à confusion avec les stries monoliformes de Siegrist au cours de la choroïdopathie hypertensive ou encore à des ruptures traumatiques de la membrane de Bruch qui surviennent dans un contexte de contusion oculaire.

**Figure 1:**
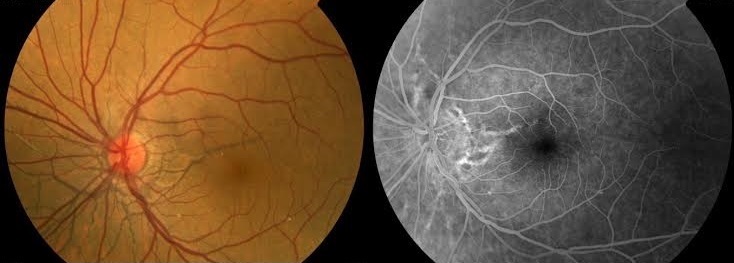
Rétinophotographie et angiographie à la fluorescéine: aspect de stries angioïdes prenant la fluorescéine

